# Gestational Diabetes Type 2: Variation in High-Density Lipoproteins Composition and Function

**DOI:** 10.3390/ijms21176281

**Published:** 2020-08-30

**Authors:** Yael Pasternak, Tal Biron-Shental, Meital Ohana, Yael Einbinder, Nissim Arbib, Sydney Benchetrit, Tali Zitman-Gal

**Affiliations:** 1 Department of Obstetrics and Gynecology, Meir Medical Center, Kfar Saba 44281, Israel; yaeli.pasternak@gmail.com (Y.P.); Tal.Biron-Shental@clalit.org.il (T.B.-S.); Nisim.Arbib@clalit.org.il (N.A.); 2 Sackler Faculty of Medicine, Tel Aviv University, Tel Aviv 6997801, Israel; yael.einbinder@clalit.org.il (Y.E.); sydneybe@clalit.org.il (S.B.); 3 Nephrology Laboratory, Department of Nephrology and Hypertension, Meir Medical Center, Kfar Saba 44281, Israel; meitalmarabi@hotmail.com

**Keywords:** GDMA2, HDL, placenta, APOA1, PON1, HUVEC

## Abstract

**Aims:** Class A2 gestational diabetes mellitus (GDMA2) has short- and long-term effects on the mother and child. These may include abnormalities of placentation, damage to endothelial cells and cardiovascular disease. This research investigated the function and composition of high-density lipoproteins (HDL) among women with GDMA2 and their fetuses. **Methods:** Thirty pregnant women were recruited during admission for delivery. The function and expression of HDL, paraoxonase1 (PON1) and apolipoprotein A1 (APOA1) in the blood samples and the placental tissue were evaluated. The effect of HDL on migration of endothelial cells was measured in vitro. **Results:** Compared to normal pregnancy (NP), APOA1 in the maternal plasma of women with GDMA2 was decreased. More APOA1 and PON1 were released from HDL of women with GDMA2, compared to NP. Placental APOA1 and PON1 were decreased in GDMA2. For endothelial cells stimulated with TNFα, HDL cell migration was decreased when cells were evaluated with NP-HDL, as compared to GDMA2-HDL. **Conclusions:** GDMA2 affects the composition and function of HDL in plasma. Changes in HDL commonly seen in GDMA2 were observed in maternal and placental samples, but not in cord samples. These results might indicate a placental role in protecting the fetus by preserving the components and functions of HDL and require further investigation.

## 1. Introduction

Gestational Diabetes Mellitus (GDM) is a classification of diabetes unique to pregnancy and is subclassified to diabetes, which requires medication to be controlled (GDMA2). It is reported to occur in as many as 5% of pregnancies in the USA and 2% to 6% in Europe [1]. GDM is associated with increased risk of adverse outcomes for both mother and fetus, during and after the pregnancy, including development of cardiovascular disease and metabolic syndrome [1,2]. 

High-density lipoproteins (HDL) exert a protective effect on the cardiovascular system by reverse cholesterol transport, atherosclerotic plaque stabilization and anti-inflammatory and anti-oxidant effects [3]. HDL subfractions are heterogeneous in size, density and protein and lipid components: apolipoprotein A1 (APOA1) accounts for approximately 70% of the total protein mass of HDL and contributes to enzyme activity, stability and paraoxonase1 (PON1) function [4,5]. Shen et al. noted that APOA1 dysfunction, decreased HDL-associated PON1 activity and their interactions are associated with the presence and severity of coronary artery disease in patients with diabetes mellitus type 2 [6]. Endothelial damage is critical in the development of cardiovascular complications and endothelial cell migration is a rate-limiting process in the repair of endothelium [7].

To our knowledge, information regarding the quantitative effects and the composition and function of HDL in women with GDMA2 and in their offspring is lacking. The characterization of changes in lipoprotein particle levels in GDMA2 may help identify lipid changes and potentially improve prediction of the risks of adverse pregnancy outcomes and postpartum metabolic diseases. 

This original study evaluated changes in maternal blood, neonatal cord blood and placentas of women with GDMA2 compared to normal pregnancy (NP). The aims of this study were: (A) to determine the quantitative and qualitative composition, as well as function of HDL among women with GDMA2; (B) to examine these aspects of HDL in the placenta and in neonatal umbilical cord blood to better understand some of the mechanisms that might put the offspring at higher risk for acquiring metabolic syndrome in the future; and (C) to evaluate whether dysfunctional HDL stimulates endothelial cell migration.

## 2. Results

### 2.1. Characteristics of the Study Population

The study included 20 women with GDMA2 and 10 with NP. Nine were balanced with insulin, 11 with oral hypoglycemic agents (10 with glyburide and 1 with metformin), each according to her healthcare provider’s preferences and consideration. Baseline clinical characteristics are shown in Table 1. None of the participants had a history of hypertension, diabetes or dyslipidemia and no GDM-related complications (retinopathy or nephropathy) were observed. HbA1c at term was averaged 5.47 ± 0.42%. None of the GDMA2 group and one of the control group had history of polycystic ovary syndrome (0% vs. 10% *p* = 0.15). Gestation was shorter in women with GDMA2 (37.9 ± 1.6 weeks vs. 39.7 ± 0.10 weeks; *p* = 0.001). There was no significant difference between the groups regarding BMI before and at the end of pregnancy, as well as in gestational weight gain. No significant differences were observed in maternal lipid profiles. There were no significant differences between insulin and oral medication use among the GDMA2 group (data not shown). Serum APOA1 levels were significantly decreased in GDMA2 (203 ± 40 mg/dL vs. 242 ± 33 mg/dL; *p* = 0.04). HDL had the strongest correlation with maternal serum APOA1 in both groups (GDMA2: r= 0.945, *p* = 0.0001, NP: r = 0.843, *p* = 0.017). No significant changes were observed in cord blood lipid profile and APOA1 between GDMA2 and NP (Table 2) and when the GDMA2 group was divided according to treatment with insulin or oral agents. 

### 2.2. Changes Observed in GDMA2 HDL Composition, APOA1 and PON1 Expression in Maternal Blood 

**Figure 1:**
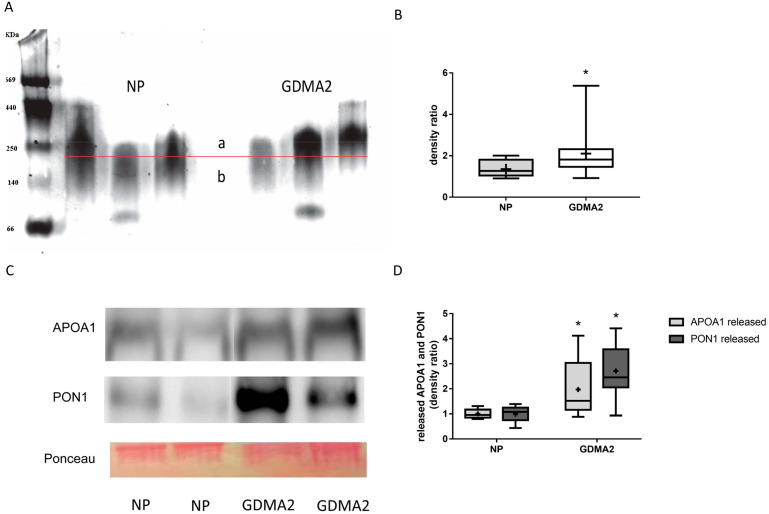
HDL composition: APOA1 and PON1 expression. (**A**) HDL from normal pregnancy (NP) (control) and GDMA2 were separated from plasma and run on native 4–12% PAGE. Band intensities above (a) and below (b) the horizontal lines drawn in the middle of each control HDL band were analyzed. (**B**) The ratio a/b was calculated for GDMA2 HDL as compared to the NP HDL (box plot of data). (**C**) HDL from GDMA2 and NP run on nondenaturing 15% PAGE followed by western blot analysis using anti-APOA1 and anti-PON1 antibodies (optical densities were normalized to Ponceau general protein stain. (**D**) APOA1 and PON1 protein expression (densitometric analysis). Data are expressed as mean ± SD of HDL isolated from 10 women with NP and 20 with GDMA2. PAGE represents two independent experiments of NP and GDMA2 each. * *p* < 0.05 compared to NP. Box plots show the five-number summary of a set of data, including the minimum score, first (lower) quartile, median, third (upper) quartile and maximum score, “+” indicates the average.

HDL was isolated from 20 women with GDMA2 and from 10 with NP. As described in the Methods Section 4.5 HDL Assays and Electrophoresis, 4–12% gradient PAGE was used to separate 20 µg of isolated HDL. HDL diameter was analyzed using multi gauge analysis software (Fujifilm) [8]. Briefly, the HDL band was divided into 2 sections (a and b in Figure 1A) by a horizontal line exactly in the middle of the control HDL. Thus, the density ratio between the 2 sections is approximately 1. When the HDL diameter increases, the ratio is anticipated to be >1, which happens when more particles appear above the line in the graph. The HDL concentration was denser among women with GDMA2, as compared to NP (2.11 ± 1.09 vs. 1.36 ± 0.43, *p* = 0.02; Figure 1B). Then, the HDL isolated from maternal blood was loaded onto nondenaturing 15% PAGE to evaluate the expression of released APOA1 and PON1. Higher amounts of APOA1 and PON1 were released in women with GDMA2, as compared to NP (1.97 ± 1.1 vs. 1 ± 0.18, *p* = 0.027 and 2.71 ± 1.0 vs. 1 ± 0.31, *p* = 0.0001, respectively; Figure 1C,D). The HDL had larger diameters and increased APOA1 and PON1 release. These changes might affect HDL anti-atherogenic activity. 

### 2.3. APOA1 and PON1 Protein Expression in Placental Tissue

**Figure 2:**
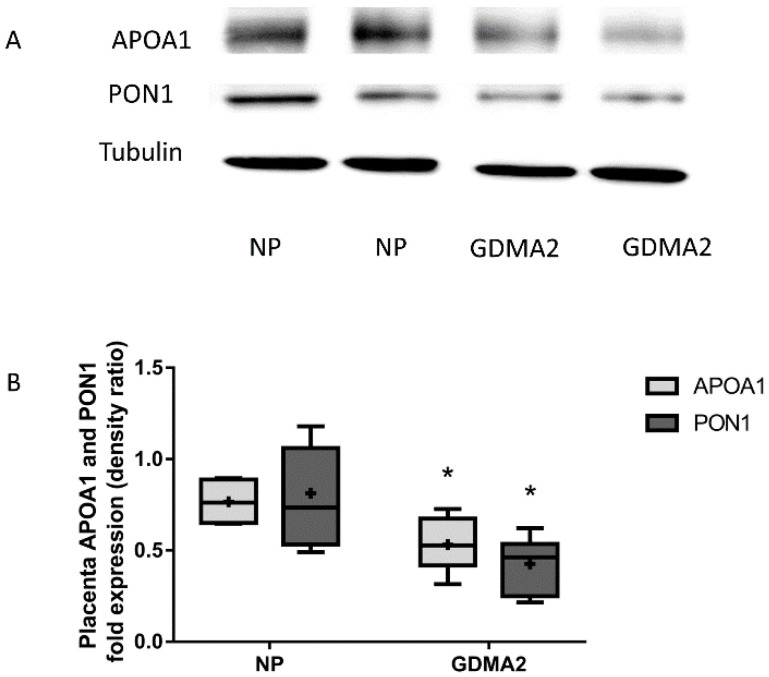
APOA1 and PON1 protein expression in placental tissue. (**A**) Protein extracted from placenta tissue run on 10% PAGE-SDS followed by western blot analysis using anti-APOA1, anti-PON1 antibodies and Tubulin (**B**) APOA1 and PON1 protein expression (densitometric analysis). Optical densities were normalized to Tubulin. Data are expressed as mean ± SD of 10 NP placenta and 20 GDMA2 placenta tissues. * *p* < 0.05 compared to NP. PAGE-SDS represents two independent experiments of NP and GDMA2, each. Box plots show the five-number summary of a set of data, including the minimum score, first (lower) quartile, median, third (upper) quartile, and maximum score, “+” indicates the average.

Placental APOA1 and PON1 protein expression were significantly decreased in GDMA2 as compared to NP (0.53 ± 0.26 vs. 0.76 ± 0.13, *p* = 0.02 and 0.42 ± 0.14 vs. 0.81 ± 0.14, respectively; *p* = 0.007; Figure 2).

### 2.4. HDL Cell Migration in HUVEC

**Figure 3:**
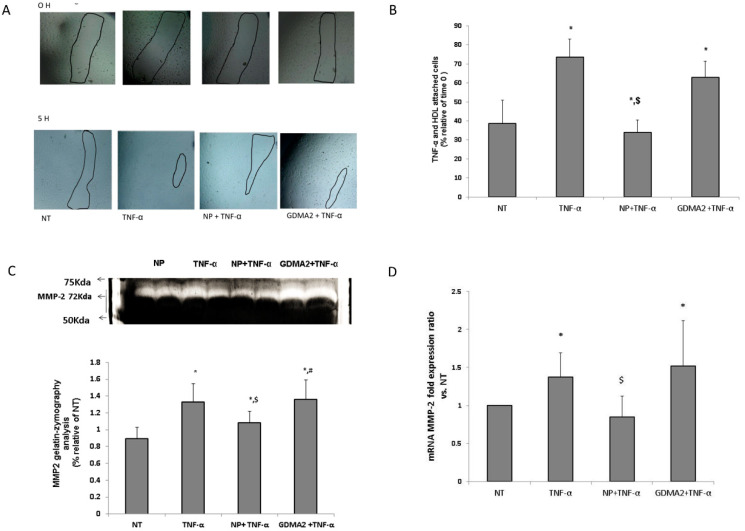
HDL and endothelial cell migration: (**A**,**B**) HDL-GDM (100 µg/mL) and HDL-NP (100 µg/mL) were added to cells for 1 h and stimulated with TNF-α (1 ng/mL) for an additional 4 h, marked in figure as NT (nontreatment), NP + TNF-α and GDM **+** TNF-α. Results were normalized to time 0. * *p* < 0.05 compared to NT, ^$^ *p* < 0.05 compared to TNF-α treatment. (**C**) After 5 h, supernatants were collected and MMPs activity was measured by gelatin zymography, relative analysis of MMP-2 results were normalized to NT. (**D**) MMP-2 mRNA fold expression ratio vs. NT. * *p* < 0.05 compared to NT, ^$^ *p* < 0.05 compared to TNF-α treatment, ^#^ *p* < 0.05 compared to NP + TNF-α treatment. Representative figures of 4 independent experiments (HUVEC from 4 different donors) including 3 technical repeats for each experiment. HDLs from different donors were used for the migration assay.

We investigated the effect of HDL in the presence of tumor necrosis factor-α (TNF-α) (as an inflammatory mediator) on human umbilical vein endothelial cells (HUVEC) migration. We assumed that the protective anti-inflammatory function of the HDL might be impaired, which could be expressed by the HUVEC migration test. Using TNF-α as a positive control showed significantly higher cell migration closure compared to negative control cells (no treatment; Figure 3A). HDL-GDMA2 achieved cell migration closure and HDL-NP reduced cell migration closure in the presence of TNF-a, as compared to negative control cells (no treatment) (Figure 3A,B). We assessed the production of matrix metalloproteinase (MMP)-2 and MMP-9, which play a critical role in the cell migration progress. The gelatin-zymography analysis showed that TNF-α and HDL-GDMA2 (in the presence of TNF-α) stimulated synthesis and activation of MMP-2. MMP-2 derived from HDL-NP had reduced gelatinolytic activity compared to TNF-α and HDL-GDMA2 (Figure 3C). Moreover, MMP-2 mRNA fold expression ratio vs. control was significantly elevated in HUVEC exposed to TNF-α (1.37 ± 0.32, *p* = 0.03) and HDL-GDMA2 (1.52 ± 0.59, *p* = 0.02) (in the presence of TNF-α), as compared to negative control cells (no treatment) (Figure 3D). No significant changes were observed in MMP-9 mRNA fold expression ratio and MMP-9 gelatin-zymography activity in all treatments (data not shown).

## 3. Discussion

This study assessed differences in HDL fractions and function among women with GDMA2, as compared to women with uncomplicated pregnancies. We found significantly decreased APOA1 levels in the maternal blood of women with GDMA2, as well as increased amounts of released APOA1 and PON1 from the HDL we isolated from their blood samples.

HDL particles have potent anti-inflammatory, anti-oxidative and antithrombotic properties due to components such as enzymes and apolipoproteins, among others. HDL lose their potential anti-atherosclerotic properties in several chronic, inflammatory conditions, including systemic oxidative stress and inflammation, diabetes and metabolic syndrome, which substantially reduce the capabilities of HDL particles and can transform them into performing pro-oxidant and pro-inflammatory activities [9,10]. 

The major apolipoprotein of HDL is APOA1, which comprises 70% of its proteome [11]. Decreased APOA1 is caused by release from HDL particles and was previously described among patients with acute coronary syndrome and noted to involve increased risk of cardiovascular disease [9]. PON1 is associated with a specific HDL subspecies and has a major role in the antioxidative activity of HDL [12]. In a recent study [8], we demonstrated that HDL from women with pre-eclampsia have decreased PON1 activity and increased APOA1 release. In the present study, we found similar patterns in APOA1 released from GDMA2-HDL. The similar results may suggest that both pregnancy complications affect the composition and function of HDL. These processes were associated with larger HDL particle diameters, which suggest impaired HDL antioxidant activity. Viktorinova et al. [13] focused on possible relationships among basic lipid parameters and lipid risk indices for cardiovascular disease, with lipid-related oxidative stress markers that reflect the actual status of lipid metabolism in patients with DM2. They found decreased PON1 and APOA1 activity in DM patients and abnormalities in the relationship of PON1 with HDL and with APOA1, potentially leading to HDL dysfunction [13]. Studies revealed that PON1 activity was decreased in women with GDM and that this may be due to increased plasma protein oxidative damage, which might create a predisposition for clinical complications in GDM [14,15,16]. Sreckovic et al. [17] found that GDM causes changes in HDL composition and is closely associated with impaired cholesterol efflux capability, as well as diminished anti-oxidative particle properties [18]. In the current study, we found larger HDL diameters among women with GDMA2. In addition, release of APOA1 and PON1 from GDMA2-HDL indicates loss of the vasoprotective properties of HDL. Decreased APOA1 levels in GDM-plasma were compatible with the findings of Timur et al. [19] who reported that women with pre-eclampsia had lower APOA1 levels than healthy controls did. 

In order to understand the mechanisms of HDL and the role of the placenta in protecting the fetus, we analyzed APOA1 and PON1 expression in placental tissue. Melhem et al. demonstrated that the placenta is an important organ, capable of producing high levels of APOs, especially APOA1 and APOE [20]. We found lower APOA1 and PON1 expression in GDM placentas compared to NP. It has been suggested that the placenta functions as a site of APOA1 synthesis, although it is not clear whether this is de novo synthesis or an accumulation from the blood stream [17]. When the maternal-fetal environment is altered, the placenta undergoes adaptive changes to ensure optimal fetal growth and development within the constraints of the prevailing intrauterine conditions. This placental plasticity creates an additional layer of protection for fetal well-being, as well as a system that potentially signals environmental conditions [17]. Eslamian et al. showed that total cholesterol, HDL cholesterol and triglyceride levels were not significantly higher in cord blood samples from neonates of mothers with GDM, as compared to controls [21]. The only difference noted in their lipid profile was higher LDL and LDL/HDL ratio, which might indicate that neonates of mothers with GDM might be predisposed to LDL hypercholesterolemia later in life [21]. Similarly, we found no significant differences in lipid profiles or APOA1 levels between cord blood samples of the GDMA2 women and controls. The changes in APOA1 and PON1 in placentas of mothers with GDMA2 vs. controls and the levels of maternal APOA1 did not appear to affect the values of fetal APOA1. These observations strengthen our knowledge regarding the vital role of the placenta in protecting the fetus.

We investigated the effect of HDL in the presence of TNF-α (as an inflammatory mediator) on HUVEC migration. We assumed that anti-inflammatory protection of the HDL might be impaired, which could be expressed by the HUVEC test. HDL in healthy subjects promotes endothelial repair by upregulating endothelial nitric oxide synthase and endothelium-dependent vasodilation. Dysfunctional HDL stimulates endothelial cell proliferation and migration [7,22]. Endothelial cell migration plays an important role in many physiological processes and in the development of diseases [23,24]. In the current study, we demonstrated the ability of HDL from NP to cause a pronounced decrease in endothelial cell migration in vitro. We found that GDMA2-HDL promoted migration of HUVEC and might promote endothelial cell dysfunction and lead to the development of cardiovascular complications in the future. 

Epithelial-mesenchymal transition (EMT) is characterized by loss of cell-cell adhesion and increased cell motility [25]. MMPs, a family of zinc- and calcium-dependent peptidases capable of degrading a wide variety of extracellular matrices, are key modulators of various biological processes, including EMT, cancer, angiogenesis, skeletal formation, inflammation and cell migration [26]. Notably, both MMP-2 and MMP-9, the two MMPs predominately expressed in endothelial cells, are crucial gelatinases that are involved in endothelial cell migration and regulate angiogenesis in endothelial cells [23,25]. We demonstrated that GDMA2-HDL achieved cell migration and stimulated activation of MMP-2 in the presence of TNF-α, while NP-HDL suppressed TNF-α-induced migration, probably through down-regulation of MMP-2. This suggests that MMPs are involved in the endothelial cell migration observed in GDMA2. 

There were some limitations to this study. The first was that the cohort was small (total of 30 participants); despite this the results were statistically significant. In addition the GDMA2 group was not managed uniformly. Although there were no differences in the demographic and clinical characteristics or in the lipid profiles of maternal and fetal blood, the subgroups were too small to have sufficient power to determine that there were no other significant differences between them.

The main strength of this study was the evaluation of HDL composition and function in all components of the maternal-fetal unit, which included maternal blood, placenta and cord blood. We suggest that the differences in HDL particles observed in GDMA2 pregnant women and in placentas but not in the umbilical cord, may protect the fetus from these harmful changes. The variations in HDL observed in our study could contribute to an increased risk of developing cardiovascular disease later in life, among women with GDMA2. These observations require additional investigation. 

Further investigation is also needed to determine whether there is a correlation between changes in HDL among women with GDMA2 and the risk of developing metabolic syndrome and cardiovascular disease. We also suggest future research to investigate the mechanisms by which the placenta prevents the fetus from acquiring the changes in HDL observed in maternal plasma and in the placentas of mothers with GDMA2.

## 4. Materials and Methods

### 4.1. Study Population

This prospective study included 20 women with GDMA2 and 10 with uncomplicated normal pregnancies (NP) who were offered enrollment in the study when they arrived at the delivery room. Inclusion criteria were normal, singleton, term delivery, with no known fetal complications. The Helsinki Committee of Meir Medical Center approved the study (no. 0132-16-MMC). All women gave written informed consent before they were enrolled in the study. 

Women were diagnosed with GDM if they had two abnormal serum glucose values [27], as reported in the American Diabetes Association guidelines [28]. Women whose glucose levels remained elevated despite changes in diet and physical activity, and were prescribed oral medication or insulin were defined as having GDMA2. Glucose levels of all women were defined by their health care providers as well-controlled, according to fasting and postprandial glucose values documented by the patient several times a day. 

Ten healthy women with uncomplicated pregnancies who were matched by age, served as the control group. Women with comorbidities such as chronic hypertension or pregnancy-related hypertension, and those with other types of diabetes (pregestational diabetes and GDMA1 managed by diet only) were excluded from the study.

### 4.2. Blood Samples

Three tubes of maternal blood and three tubes of neonatal cord blood were taken by venipuncture for total HDL extraction and APOA1 analysis. Plasma was harvested using low speed centrifugation (3000× *g* for 10 min at 4 °C). Plasma samples were stored at −80 °C before analysis. Serum was harvested after low speed centrifugation (3000× *g* for 3 min at 4 °C) and stored at −80 °C before analysis.

### 4.3. Placental Biopsies

Placenta samples were taken under sterile conditions, 1 cm^2^ from the area midway between cord insertion and the edge of the placenta. Each specimen was cut into five mm^3^ pieces and kept on ice to prevent RNA degradation. They were frozen at −80 °C within 20 min of delivery.

### 4.4. APOA1 Concentration

The APOA1 concentration in maternal and cord blood from GDMA2 and NP were determined using the Tina-quant Apolipoprotein A-1 kit, (Cobas Integra Analyzers, Roche Diagnostics, Indianapolis, IN, USA).

### 4.5. HDL Assays and Electrophoresis

Discontinuous density gradient ultracentrifugation (*d* = 1.006–1.25 g/mL) was used to isolate HDL from the plasma samples, as described previously [12]. The modified Lowry protein assay kit (Thermo Scientific, Rockford, USA) was used to determine HDL protein concentration. Twenty micrograms of isolated total HDL were separated according to hydrodynamic diameter by nondenaturing, 4–12% gradient polyacrylamide gel electrophoresis (PAGE) (4–12% Tris-glycine gel; Bio-Rad Laboratories, Carlsbad, CA, USA), as described previously [12]. The globular proteins, 17 nm thyroglobulin, 12.2 nm ferritin, 10.4 nm lactate, 8.2 nm dehydrogenase and 7.1 nm albumin, were used as references (high molecular weight calibration kit, Amersham Pharmacia Biotech, Buckinghamshire, The UK). In addition, 20 µg of isolated total HDL were separated on nondenaturing 15% PAGE and electrophoretically transferred to a nitrocellulose membrane in order to analyze APOA1 and PON1 expression.

### 4.6. Placenta Protein Extraction

A mixture of 400 µL lysis buffer (25 mM tris (pH 7.5), 1% Triton X-100, 0.5 mM EDTA, 150 mM NaCl, 10 nM NaF, 10 μg/mL leupeptin, 10 μg/mL pepstatin, 200 μg/mL PMSF and 1:100 phosphatase inhibitor) was used to homogenize 40 mg of frozen placental tissue. The centrifuge tube was agitated at 4 °C for 2 h. The BCA^TM^ protein assay kit (Thermo Scientific, Rockford, IL, USA) was used to determine protein concentration (following the manufacturer’s instructions). Total protein (50 μg) from all 30 placentas was separated on 10% SDS-PAGE and transferred to a nitrocellulose membrane using electrophoresis.

### 4.7. Western Blot

A standard western blot technique using 1:500 anti-APOA1 polyclonal antibody (Millipore, Temecula, CA, USA), 1:500 anti-PON1 monoclonal antibody (Abcam, Cambridge, MA, USA) and 1:8000 anti-Tubulin (Sigma-Aldrich, Detroit, MI, USA) was used to evaluate APOA1, PON1 and Tubulin protein expressions in HDL fractions and placental tissue. The enhanced chemiluminescent reporter system (Biological Industries, Bet Ha’emek, Israel) was used to visualize the bound antibody. Protein expressions were quantified using LAS-3500 (Fujifilm, Tokyo, Japan). The optical densities were normalized to Ponceau stains or to antiTubulin.

### 4.8. Cell Culture and Incubation 

Mothers provided informed consent for use of umbilical cords, which were obtained from the labor and delivery department of Meir Medical Center in Kfar Saba, Israel [29]. The Helsinki Committee of Meir Medical Center approved the study (no. 0074-11-MMC). Fresh human umbilical vein endothelial cells (HUVEC) were isolated and grown in M-199 medium that had been supplemented with 5 U/mL heparin, 25 µ/mL endothelial mitogen (Biomedical Technologies, Inc., Stoughton, MA, USA) and 20% FCS, 100 U/mL penicillin and 100 µ/mL streptomycin (Biological Industries, Beit Ha’emek, Israel). HUVEC were used at passage 3–4 for experiments and were pre-incubated with HDL (100 µg/mL, 1 h) and stimulated with tumor necrosis factor-α (TNF-α) (1 ng/mL, 4 h), as previously described (with modifications).

### 4.9. Cell Migration (Scratch Test)

Endothelial cells (5  ×  10^3^) were placed in 96-well plates and allowed to reach an approximately 70% confluent monolayer. HDL-GDM (100 µg/mL) and HDL-NP (100 µg/mL) were added to cells for 1 h and stimulated with TNF-α (1 ng/mL) for an additional 4 h to induce an inflammation condition. Plates were scratched and wound closure was monitored immediately afterward (time 0) and 5 h later. Areas were measured using ImageJ software (National Institutes of Health, Bethesda, MD, USA). Four independent experiments (HUVEC from 4 different donors) including 3 technical repeats for each experiment. HDL from different donors were used for the migration assay. 

### 4.10. Gelatin Zymography

Media (40 μL) from HUVEC scratch tests (as described above) were electrophoresed at nonreducing conditions in 10% polyacrylamide gel containing 0.2% gelatin as an MMP substrate. The gels were washed in 2.5% Triton X-100 and incubated overnight in 50 mM Tris-HCl (pH 7.5) and 5  mM CaCl_2_. First Coomassie blue staining and then antistaining with 20% methanol, and 7% acetic acid in double distilled water, allowed results in clear lysis zones to be seen against a blue background. mMatrix metalloproteinase (MMP)-2 and MMP-9 were determined from intermediate and active MMP-2 and MMP-9 by zymography, depending on the molecular weight. Optical densities of the clear zones revealed by the enzyme activities were measured as arbitrary units using the LAS3000 Image reader (Fujifilm). The results were normalized to background values using the Multi-gauge V3.0 program (Fujifilm). 

### 4.11. Real Time Polymerase Chain Reaction (PCR)

MasterPure RNA purification kit (EPICENTRE, Madison, WI, USA) was used to extract total RNA from HUVEC obtained from the scratch test, in accordance with the directions provided in the kit. The high capacity cDNA reverse transcription kit (Applied Biosystems, Inc., Foster City, CA, USA) was used to reverse transcribe 1 µg RNA into single-stranded DNA.

Real-time PCR was performed to validate the expression pattern of matrix metalloproteinase (MMP)-2 forward: 5′CAAGGACCGGTTTATTTGGC3′, reverse: 5′ATTCCCTGCGAAGAACACAGC3′), MMP-9 (forward: 5′CCTGGGCAGATTCCAAACCT3′, reverse: 5′CAAAGGCGTCGTCAATCACC3′) and glucuronidase beta (GUSB) as reference gene (forward: 5′CAATACCTGACTGACACCTCCAGTA3′, reverse: 5′TGGTGGGTGTCGTGTACAGAAGT3′).

### 4.12. Statistical Analyses

The sample size was calculated based on an assumption of a 2-fold change in the composition of HDL, alpha of 5% and power of 80%. All data are expressed as mean ± standard deviation (SD) or median (range). The Shapiro-Wilk test was used to test for normality. Paired *t*-test or Wilcoxon signed-rank test for nonparametric data, were used to evaluate changes in the protein expression of PON1 and APOA1 and for cell migration experiments (as appropriate). Pearson or Spearman correlations were used to analyze the relation between APOA1 and HDL using Fisher r-to-z transformation. *p*-values < 0.05 were considered significant. Data and Box Plots were analyzed using SPSS-25 (IBM Corporation, Armonk, NY, USA). Graphs were done using GraphPad Prism version 7.00 for Windows (GraphPad Software, La Jolla California USA, www.graphpad.com).

## Figures and Tables

**Table 1:** Demographic and clinical characteristics of pregnant women with gestational diabetes mellitus type 2 (GDMA2) and of healthy women during an uncomplicated pregnancy. HDL: high-density lipoproteins.

Characteristic	GDMA2 (N = 20)	Normal Pregnancy (N = 10)	*p*-Value
Age (years)	33.2 ± 5.2	28.4 ± 6.0	0.38
Gestational age (weeks)	37.9 ± 1.6	39.7 ± 1.0	0.001
Body mass index (kg/m^2^)	29.8 ± 11.2	30.6 ± 4.4	0.79
Systolic blood pressure (mmHg)	116.6 ± 7.2	116.4 ± 8.2	0.94
Diastolic blood pressure (mmHg)	74.6 ± 9.7	73.0 ± 10.9	0.70
Total serum cholesterol (mg/dL)	226.7 ± 51.8	249 ± 54.5	0.29
Serum triglycerides (mg/dL)	238 ± 105.6	239 ± 91	0.99
Serum HDL cholesterol (mg/dL)	61.0 ± 15.6	66.9 ± 12.1	0.28
Serum LDL cholesterol (mg/dL)	125.8 ± 52.0	136.4 ± 45.4	0.61
Fasting glucose (mg/dL)	94.7 ± 16.12	84.1 ± 9.9	0.06
APOA1 (mg/dL)	203 ± 40	242 ± 33	0.04

Data are expressed as mean ± SD.

**Table 2:** Lipid profile of umbilical cord blood from offspring of women with gestational diabetes mellitus type 2 (GDMA2) and from normal pregnancies.

Characteristic	GDMA2 (N = 20)	Normal Pregnancy (N = 10)	*p*-Value
Total serum cholesterol (mg/dL)	58.3 ± 13.1	54.3 ± 17.0	0.54
Serum triglycerides (mg/dL)	40.9 ± 15.2	41.1 ± 10.14	0.96
Serum HDL cholesterol (mg/dL)	27.0 ± 10.1	24.3 ± 11.3	0.55
Serum LDL cholesterol (mg/dL)	22.4 ± 6.2	21.9 ± 8.6	0.86
APOA1 (mg/dL)	84.0 ± 12.0	76 ± 12	0.16

Data are expressed as mean ± SD.
